# Increasing Awareness and Early Detection of Common Skin Diseases in Indonesia Through an mHealth App: Protocol for an Awareness and Acceptability Study and Randomized Controlled Trial

**DOI:** 10.2196/64057

**Published:** 2025-09-08

**Authors:** Ulfah Abqari, Muhammad Atoillah Isfandiari, Jan Hendrik Richardus, Ida Korfage

**Affiliations:** 1 Department of Public Health Erasmus MC University Medical Center Rotterdam The Netherlands; 2 NLR Indonesia Jakarta Indonesia; 3 Faculty of Public Health Airlangga University Surabaya Indonesia

**Keywords:** community health awareness, digital health intervention, early detection education, health application usability, mHealth, skin diseases, leprosy, Indonesia

## Abstract

**Background:**

Various media are used to enhance public understanding about diseases. While mobile health apps are widely used, there is little proof for using such apps to raise awareness of skin diseases.

**Objective:**

We intend to develop an app, called DEDIKASI-app, to raise awareness of skin diseases, including leprosy. The study will explore baseline awareness, assess the app’s acceptance by community members and health care workers, and evaluate its effectiveness in enhancing awareness about skin diseases.

**Methods:**

The study will be conducted in four phases: (1) development of DEDIKASI-app, (2) questionnaire development for an awareness study, (3) acceptability testing, and (4) effect measurement of DEDIKASI-app. We will adopt design thinking methodology to develop the app, involving systematic reviews, expert consultations, focus group discussions, and validation of the questionnaire on skin disease awareness. We will recruit 50 members of the community for the awareness and acceptability study and 1 health care worker per community health center to assess their perception of the app. A pilot study will assess the acceptability of DEDIKASI-app among community members and health care workers based on various constructs, with responses categorized as positive, negative, or undecided. The validity and reliability of a newly developed questionnaire on skin disease awareness will be tested, with validity results analyzed qualitatively and reliability measured using Cronbach α. The effectiveness of DEDIKASI-app in improving community awareness will be evaluated through a randomized controlled trial, using total scores, means, and SDs for control and intervention groups. Statistical significance of awareness level changes will be determined by delta change (*P* value), with *P*<.01 considered significant.

**Results:**

This study received ethical approval from the Ethics Review Board of the Faculty of Public Health, Universitas Airlangga (160/EA/KEPK/2023) and was registered in the Indonesia Clinical Research Registry (INA-O8EX278). Funding for the field research was secured in the period of May 2022-December 2024 from NLR Indonesia and Erasmus University Medical Center. As of manuscript submission, phase 1 (app development) and phase 2 (questionnaire development) have been completed. Data collection for the randomized controlled trial has just finished and data analysis is ongoing, with publication of the study results expected in late 2025.

**Conclusions:**

Innovative approaches are required to enhance awareness in the community. This study will introduce new tools and insights to address the limited knowledge about detecting skin problems.

**Trial Registration:**

INA-CRR Indonesia Clinical Research Registry INA-O8EX278; https://tinyurl.com/ms3k5yvn

**International Registered Report Identifier (IRRID):**

DERR1-10.2196/64057

## Introduction

Skin diseases are common all over the world. They can range from non–life-threatening conditions such as acne, contact dermatitis, benign tumors, atopic dermatitis (eczema), and psoriasis to more serious conditions such as autoimmune diseases and skin cancers. A particularly important group of skin diseases, categorized as neglected tropical diseases (NTDs), afflict disadvantaged communities in low- and middle-income countries [1,2]. These include Buruli ulcers, cutaneous and postkala azar dermal leishmaniasis, lymphatic filariasis, onchocerciasis, mycetoma, yaws, chromoblastomycosis and other deep mycoses, scabies and other ectoparasites, and leprosy [2]. Many of these conditions can cause lasting physical and mental complications and are associated with stigma. Leprosy is a typical example of such an NTD, with nearly 200,000 new cases reported worldwide in 2021 [3].

Detection delay is an important factor associated with disabilities in leprosy [4]. The stigma associated with leprosy, often exacerbated by a lack of understanding, challenges early detection considerably [5,6]. Stigmatization leads people to hide their condition, and this avoidance behavior results in delayed diagnosis and treatment [7-10]. The combined lack of knowledge and stigma creates barriers for prompt implementation of case finding and adherence to treatment protocols, undermining the overall effectiveness of leprosy control [7,11-13]. A pivotal element of timely detection is fostering self-awareness among populations at risk. Increasing public awareness on the relevance of early detection holds the potential to shift attitudes and behaviors toward increased health-seeking behavior [14]. This holistic approach should also target dispelling myths and misconceptions tied to leprosy. Furthermore, reinforcing health care systems is crucial to ensure streamlined diagnostic and treatment processes, while guaranteeing respectful and sensitive care for affected individuals [15-17].

National programs use information, education, and communication media to improve the knowledge and awareness of the community about health conditions and diseases. Both printed and electronic media are used widely for showcasing innovative approaches used to disseminate health-related messages [18]. The surge in mobile phone use has allowed health care support through mobile health (mHealth), a subset of eHealth. mHealth involves the application of mobile computing and communication technologies in health care and public health, commonly realized through mobile applications [10,18]. In 2018, the World Health Organization introduced an eHealth guidance book advocating mobile apps to enhance health services and achieve universal health coverage [19]. Aligning with this initiative, the Indonesian Ministry of Communication and Information initiated the Smart Health Initiative to digitize health services in the country [20]. The distribution of apps, however, varies across disease conditions and intended goals. Moreover, there is currently no evidence supporting the use of an app for raising awareness about early leprosy detection [21].

The proposed study entails the creation of a 2-way integrated mobile app known as DEDIKASI-app (“dedication” in English and would be application-based early skin detection translated from the Indonesia abbreviation), catering to both users and health care workers (HCWs). The application aims to raise awareness about early skin detection, encompassing possible leprosy cases and other prevalent skin conditions. Because of the stigma toward leprosy in the community, this study extends its scope to include common skin diseases within the app.

The study aims are as follows: (1) to develop a mobile app (DEDIKASI-app) as an awareness-raising strategy in the community for early signs of common skin diseases, (2) to develop a skin diseases awareness questionnaire, (3) to provide insight into the acceptability of DEDIKASI-app to the community and HCWs, and (4) to assess its effectiveness in improving community awareness of common skin symptoms.

## Methods

### Overview

**Figure 1:**
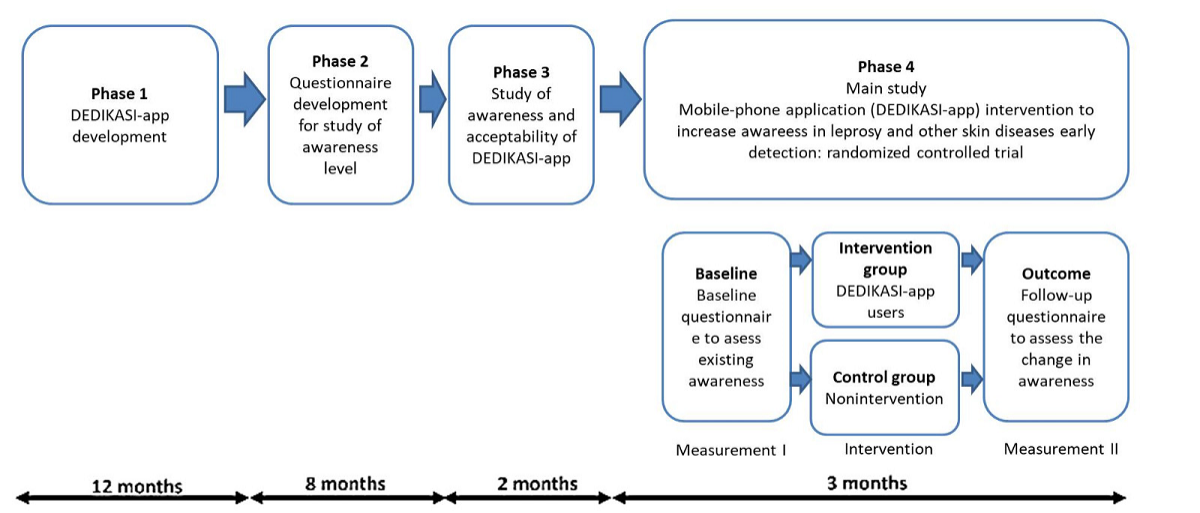
Flowchart of the 4 phases of the study.

The study will be conducted in four phases: (1) development of the DEDIKASI-app mobile app, (2) development of a questionnaire to measure the level of awareness in the community of common skin diseases, (3) an awareness study and acceptability test of DEDIKASI-app, and (4) a randomized controlled trial of DEDIKASI-app. The phases of this study are outlined in Figure 1.

### Phase 1: Development of DEDIKASI-app

We will apply a comprehensive approach to create an effective and user-friendly platform for common skin disease detection and awareness promotion. The platform is called DEDIKASI-app, an acronym of deteksi dini kusta berbasis aplikasi*,* which translates to application-based early skin detection. DEDIKASI literally means “dedication,” portraying the dedication to achieve leprosy elimination by increasing awareness and early diagnosis using this DEDIKASI-app.

DEDIKASI-app is a 2-way platform aimed at the general community (DEDIKASI-P) and HCWs (DEDIKASI-HCW) within community health centers (CHCs, puskesmas in Indonesia). The front end of the platform is openly accessible to the public while the back end is only accessible to HCWs functioning on Android devices with or without internet connectivity. While the front end allows offline reading of information, active tasks, such as skin self-detection and uploading, require internet access. Similarly, the back end can function offline for reading, but internet connectivity is essential for notifications and responses.

DEDIKASI-P will include an animated anatomy feature and a detailed catalogue of skin symptoms to assist users in self-assessment by offering valuable information. After completing the self-assessment, HCWs can review the results through DEDIKASI-HCWs, enabling internet-based consultations or scheduling in-person appointments.

Before developing the app, we conducted a systematic literature review of studies on mHealth apps and interventions to explore strategies and tools for promoting mHealth interventions aimed at promoting early detection and increasing awareness of chronic diseases [22]. This review helped identify various approaches to enhance early detection and awareness while also incorporating strategies to maintain user engagement. Additionally, expert consultations were carried out to design a user-friendly interface accessible to all community members, regardless of their educational background.

The mobile apps will be developed by identifying suitable design architecture and an interface aligned with its intended purpose. This step will entail outlining the overall requirements for app development, with content generated in the Indonesian language. DEDIKASI-app will be entirely self-developed, including its visual design and animations, ensuring that no copyrighted materials from other apps or owners are used in its development. Design thinking methodology [23], a human-centered approach, will be adopted to delineate the standardized process used in crafting the app. This methodology focuses on user needs and considers various stakeholders’ perspectives, involving HCWs closely in the development process [23].

We emphasize that DEDIKASI-app is not intended to replace the role of health care professionals in diagnosing common skin diseases. Instead, the app will serve as an interactive and communicative tool for raising awareness. The app aim to stimulate early common skin disease detection, particularly of leprosy, preventing severe physical and mental outcomes and stigma. Additionally, the users will be encouraged to seek help from HCWs by using DEDIKASI-app when they identify potential skin symptoms. Given the social isolation and stigmatization faced by individuals with leprosy, the app’s internet-based services offer empowerment and privacy, allowing for self-testing and reducing stigma-related fears [24-26]. However, the role of health care professionals remains crucial in case finding, diagnosis, and management of leprosy.

Based on prior studies, strategies will be selected to attract users, such as attractive animations, tailored messages, and rewards (eg, receiving a shopping voucher). Visual appeal, ease of use, social support, and trust are factors that encourage health app usage [27-29]. The app will feature an anatomy animation and a comprehensive list of skin symptoms aimed to aid the users in self-assessment by providing helpful information.

Although the main objective of DEDIKASI- app is to increase awareness about leprosy, given the stigma about leprosy, we decided to expand the context of the app to common skin diseases.

DEDIKASI-app will be developed by a recognized digital health agency in Indonesia called Databiota. The Databiota team will use Android Studio to develop the app’s software, with initial user evaluation during app prototyping. A navigating map (site map and screen flow diagram) will be applied for modeling graphs to describe the interface map. Version control and update tracking will be reported once the app is finalized and the trial begins.

App development will be funded by NLR Indonesia. The app will be owned by the research group on Tropical Diseases, Infectious Diseases and Herb in the Faculty of Public Health Universitas Airlangga in Surabaya, Indonesia.

### Phase 2: Development of a Questionnaire for Measuring Awareness of Common Skin Diseases in the Community

The literature review showed the absence of specific instruments for measuring the level of awareness in the general community of early symptoms of common skin diseases. Thus, in this second phase of the study, we aim to develop a questionnaire for measuring awareness in the general community, which will assess common skin disease knowledge, common skin disease prevention, and the intention to seek health services.

The questionnaire will be developed in 2 steps. The first step involves conducting a literature review, consultation with professional experts, and focus group discussions with persons with and without knowledge or expertise in leprosy. Questions will initially be developed in English based on expert judgement and checks by English language experts. Because the study population is Indonesian, the second step will involve translating the questionnaire into the Indonesian language.

The validity and reliability study for the developed questionnaire will be conducted in the subdistrict of Batumarmar, Pamekasan District, East Java Province, ensuring the questionnaire accurately measures the awareness of the community regarding skin diseases and produces consistent results over time and different respondents. The Batumarmar area was chosen because of its variety of geographic and community characteristics, which include rice fields, mountains, and residential areas in both urban and rural settings. Study participants will include experts in leprosy and behavioral sciences (the study supervisor), local key informants, and members of the general community.

The eligibility criteria for the validity and reliability study are as follows: (1) residents living in Batumarmar subdistrict and Pamekasan District; (2) individuals aged 18-65 years; (3) individuals willing to participate and provide informed consent; and (4) individuals able to understand and communicate in Indonesian and Madurese languages. Aside from the results of the validity and reliability test, data on demographical characteristics, such as gender, age, educational level, and occupation, will be included for analysis.

### Phase 3: Awareness Study and Acceptability Test of DEDIKASI-App

#### Awareness Study

Implementation of the awareness study in the general community will follow a cross-sectional mixed-method approach. The population being studied is the general community in Pamekasan District. Fifty respondents will be recruited from 4 CHCs that were randomly selected based on their representativeness of urban and rural areas in Pamekasan District. Two CHCs were chosen to represent urban areas (Teja and Proppo), while the other 2 represent rural areas (Sopa’ah and Galis). Recruitment of respondents will be conducted using a simple random sampling technique. The number of respondents is based on a minimum sample size proposed by Terwee et al [30] for the measurement properties of health status questionnaires.

The eligibility criteria for respondents are as follows: (1) residents living in the 4 CHCs in Pamekasan District, (2) individuals aged 18-65 years, (3) individuals willing to participate and provide informed consent, and (4) individuals able to understand and communicate in Indonesian and Madurese languages.

#### Acceptability Test

In parallel with the awareness study, acceptability testing for DEDIKASI-app will be conducted with the same 50 respondents selected for the awareness study. This study aims to measure the acceptability level of the community toward DEDIKASI-app and to assess their perception toward its use. In addition, 21 HCWs (1 from each of the 21 CHCs) will be included in an acceptability test of the DEDIKASI-HCW app to assess their perception of its use. We will include HCWs who actively coordinate with leprosy supervisors at the district health office level.

DEDIKASI-app will be shown to all participants. Community and HCWs’ acceptability, defined as the degree to which users are willing to adopt and use a new technology influenced by key psychological and behavioral factors, will be conducted using a survey based on the technology acceptance model (TAM). The TAM is widely adopted to assess the acceptability of health technologies within the 4 constructs of perceived usefulness, perceived ease of use, attitude, and behavioral intention to use [31,32]. Based on previous studies [28,29], the following 4 constructs will be added: security and privacy, technology literacy, quality of information, and benefit. For the TAM questionnaire with these added constructs, we will not conduct a separate validation, as the reasons for including these constructs have already been established and validated in prior studies. However, in the current study, we will train the research assistants to carefully observe whether the respondents understand the questions during administration to ensure clarity and comprehension. A 5-point Likert scale will be used to quantify the responses.

### Phase 4: Randomized Controlled Trial of the Effectiveness of DEDIKASI-App to Increase Early Detection of Skin Diseases Including Leprosy

The fourth phase is an randomized controlled trial aimed to assess the effectiveness of DEDIKASI-app as a strategy to increase awareness and early detection of leprosy and other common skin diseases.

The sample size will be calculated using the following formula for hypothesis testing for 2 population means (2-sided test) [33]:

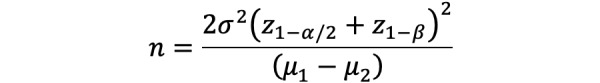



The following values will be used: level of significance (α)=5%; power of the test (1-β)=90%; population SD (σ)=5; and population variance (σ^2^)=25. The population mean (µ_1_) will be obtained after the awareness study in the second phase; as there was no previous study available, µ_2_ represents the anticipated population mean of the intervention group. Sample size will be calculated afterward; however, we will expect to obtain 600 participants.

The study population is the general community located in 21 CHCs of Pamekasan District. The participating CHCs have already been randomly allocated to either the intervention or control group using a computer-generated sequence by an independent statistician. The intervention group comprises Tlanakan, Bandaran, Pademawu, Sopaah, Galis, Larangan Bedung Kadur, Batumarmar, Teja, and Kowel, and the control group comprises Larangan, Talang, Proppo, Panaguan, Palengaan, Pakong, Waru, Tampojung Pregi, Pasean, Pengantenan, and Bulangan Haji. Allocation concealment was ensured by using sequentially numbered, sealed, opaque envelopes, opened only after enrolment of each cluster. Research assistants will enroll participants through door-to-door recruitment*.* To reduce information contamination, we will ensure geographical separation of intervention and control CHCs where feasible. Pamekasan District, located on Madura Island in East Java Province, covers an area of approximately 792 square kilometers, with a population density of around 850 people per square kilometer. The district is divided into 13 subdistricts, with substantial distances between them; for example, the distance between Batumarmar (a northern subdistrict) and Proppo (a central subdistrict) is approximately 25 kilometers, often requiring over an hour’s travel due to rural road conditions. Given this geographic spread and limited daily intervillage interactions, the risk of information spillover between intervention and control clusters is considered minimal. Baseline and postintervention surveys will be conducted within a short period of a fixed 4-week window across all clusters to minimize variation due to external influences such as health campaigns or seasonal trends. Data collectors will follow the arranged schedule based on the study procedure. For eligibility to participate in the study, participants must be a resident of Pamekasan District, especially in the working area of the 21 CHCs; aged 18-65 years; willing to participate in the study and provide informed consent; and an android or smartphone user. The final criterion might not apply to the control group.

Data regarding the effectiveness of DEDIKASI-app as a strategy to increase awareness and early detection of leprosy and other common skin diseases will be collected using the validated questionnaire that will be developed in phase 2.

### Statistical Analysis

Results of validity and reliability testing of the community awareness questionnaire developed in phase 2 will be analyzed using both descriptive and analytical statistics. Demographic data will be analyzed descriptively, while the validation and reliability of the questionnaire will be assessed statistically using Pearson correlation and Cronbach α, respectively. Content and face validity will be qualitatively used to avoid question item ambiguity, ensure clarity and relevancy, and ensure that the questions are not judgmental, intrusive, or distressing to target participants [34]. The questionnaire will be administered through an interview setting, with all selected respondents interviewed at 2 points: a preintervention interview and a postintervention interview conducted approximately 4 weeks later (during the intervention study in phase 4).

During the third phase, the questionnaire developed in phase 2 will be used to collect the data on awareness in the general community. All responses to the questions will be given numerical scores based on correct or positive answers. The total score will be calculated, while the mean range will be used to illustrate the distribution and variation of scores. To measure the impact of the intervention, the change in scores (delta change) between preintervention and postintervention assessments will be calculated for each group. The Wilcoxon signed-rank test will be used to compare paired preintervention and postintervention scores within each group. A *P* value of <.01 will be considered statistically significant, indicating improvements in respondents’ knowledge, prevention practices, and health-seeking behavior.

The acceptability test for DEDIKASI-app will be conducted during the third phase, using a survey based on the TAM. A Likert scale will be used as a scoring system. The results from both community members and HCWs will be descriptively analyzed and presented using a frequency table.

Data from the randomized controlled trial in the fourth phase will be analyzed using SPSS version 21 (IBM Corporation). We will conduct a descriptive analysis by calculating the either the mean or median values of the awareness scores and the count of reported common skin disease cases through DEDIKASI-app. The choice between mean or median will depend on the distribution of the scores. We will also use a box plot diagram to visually compare the measurements between the 2 groups.

For assessing the effectiveness of DEDIKASI-app as an intervention for enhancing awareness compared to the control group, we will apply an independent sample *t* test if the data exhibit a normal distribution. If the data distribution is not normal, we will use the Mann-Whitney test. All statistical analyses will be performed at a significance level of .05, with a 95% CI (or using the IQR if medians are used).

Missing data will be described and explored. We acknowledge that incomplete case analysis can introduce bias and will consider appropriate imputation techniques during final analysis depending on the extent and pattern of missingness. We will include the outcomes of all participants (intention-to-treat), regardless of the extent the intervention group used DEDIKASI-app.

### Ethical Considerations

Ethical clearance for the study (all 4 phases) has been sought from the Ethical Review Board of the Faculty of Public Health Universitas Airlangga, Surabaya, with registration number 160/EA/KEPK/2023. The trial in phase 4 has been registered to the INA-CRR, the trial study registry of the Ministry of Health, Republic of Indonesia (INA-O8EX278). Paper-based informed consent will be asked from all participants. Participants will be informed during the consent process whether they will be allocated to the intervention or control group. As such, participants will be aware of whether or not they will be using DEDIKASI-app. This may have influenced their expectations.

All participant data collected via DEDIKASI-app will be stored securely with encryption and password protection. Only authorized HCWs will have access to identifiable information through the HCW interface. Study participants will be informed of data confidentiality, and in case of health concerns, they may be referred to their nearest CHC for follow-up. Research assistants and HCWs involved in the study will receive training on privacy, communication protocols, and referral procedures.

## Results

### Current Progress

This study received ethical clearance from the Ethics Review Board of the Faculty of Public Health, Universitas Airlangga (160/EA/KEPK/2023) and is registered in the Indonesia Clinical Research Registry (INA-O8EX278). Field research was funded by NLR Indonesia and Erasmus MC for the period of May 2022 to December 2024. At the time of manuscript submission, phase 1 (DEDIKASI-app development) and phase 2 (questionnaire validation) had been completed. Data collection for the randomized controlled trial has recently concluded, and data analysis is currently underway. Final study results are expected to be published by the end of 2025.

### Phase 1: Development of DEDIKASI-App

The app will be developed following the design thinking methodology, incorporating literature reviews, expert consultations, and focus group discussions. The final version of the app will be validated by a panel of health care professionals and community members to ensure its usability and relevance.

### Phase 2: Awareness Study and Questionnaire Development

A questionnaire will be developed and validated to measure awareness levels of common skin diseases. It is expected that initial results will show varying levels of awareness among different demographic groups, with the highest awareness likely among HCWs. Statistical validation will be conducted to confirm the reliability and accuracy of the questionnaire.

### Phase 3: Acceptability Testing

The acceptability study will assess whether DEDIKASI-app is well-received by both community members and HCWs. Usability scores will be measured, and key concerns such as the need for localized content and ongoing updates will be identified.

### Phase 4: Randomized Controlled Trial

The intervention study will evaluate whether there is a statistically significant increase in awareness and early detection behaviors among the intervention group compared to the control group. It is expected that participants using DEDIKASI-app will be more likely to seek professional health care assistance for skin conditions. Additionally, skin conditions reported by members of general public in the intervention group will be analyzed.

Although this is a low-risk, behavioral study, we will monitor for any unintended or unexpected events such as technical issues, privacy concerns, or participant distress resulting from the use of DEDIKASI-app. These events, if they occur, will be documented and reviewed.

## Discussion

### Anticipated Findings

Innovations are needed for improving awareness in the general community of symptoms of skin diseases, especially of those that are contagious or need timely treatment to prevent future complications. This is particularly the case for leprosy, in which disease elimination and disability prevention depend on early case detection and treatment. Aligned with World Health Organization guidance in applying digital health innovations, mobile phone apps are expected to improve health services in the community and place us on the road to achieving universal health coverage [35]. Through our proposed study, we will develop a mobile phone app aimed to increase awareness in the community of common skin diseases, including leprosy. DEDIKASI-app aims to address gaps in awareness and early detection of skin-related NTDs, including leprosy and other common skin diseases, by leveraging mHealth technology in a low-resource setting. We anticipate that it will be acceptable to both community members and HCWs and will lead to measurable improvements in awareness and early detection. The integrated approach of DEDIKASI-app broadens the scope of the app and simultaneously addresses the stigma attached to leprosy by not singling out just one disease, possibly discouraging its use by the general public.

Unlike previous mHealth interventions that focus on treatment adherence or symptom tracking, this app will integrate culturally tailored content and localized language support to target stigma and misinformation [25,26].This study will produce newly developed instruments and add insights into the level of knowledge regarding awareness of common skin diseases. The study will also provide insight into the acceptability and effectiveness of DEDIKASI-app for both the community and HCWs.

The app could be adapted to other NTDs or used in other Indonesian provinces with future validation. Results will be disseminated via journal publication, stakeholder workshops, and presentations to health policymakers and nongovernmental organizations.

### Strengths and Limitations

This study represents a pioneering effort in Indonesia as it is a novel study assessing awareness levels of skin diseases among the community and HCWs. As such, it is expected to fill a significant gap in existing knowledge. The study’s strengths include a comprehensive design, which will use design thinking, the TAM, and a multiphase development and validation process. The integration of mobile technology in DEDIKASI-app serves as a key strength, enhancing accessibility and engagement in health education. The study uses a randomized controlled trial design, ensuring rigorous evaluation of the app’s effectiveness. Additionally, the current study will monitor to which extent participants will actually reach out to CHCs with questions about their skin.

However, there are several limitations to consider. The first limitation includes potential challenges with digital literacy, limiting generalizability to similar rural populations across Indonesia with large characteristic variation. The study is conducted only in Pamekasan District, Madura Island, East Java, which has specific social and geographical characteristics, potentially limiting the generalizability of the findings. Although this study is limited to community members in Pamekasan District, the design of DEDIKASI-app allows for future use in other settings. The findings will be generalizable to similar rural or underserved populations in Indonesia or other low-resource areas. However, additional evaluation will be needed before applying this intervention in urban or higher-literacy populations or through fully web-based environments. Second, this study may face limitations common to eHealth interventions, including the inability to blind participants, which may introduce expectation bias. This limitation will be mitigated by ensuring anonymity, using validated questionnaires, and cross-referencing with objective measures where possible. Third, while mobile technology enhances accessibility, digital literacy and internet connectivity issues may affect the intervention’s reach and effectiveness. Providing user training sessions and offering alternative offline materials will help ensure broader usability and impact. Additionally, participants’ awareness of being observed or assessed may influence their behavior (the Hawthorne effect) [36]. Lastly, although multiple outcomes will be examined, this pilot study is not powered to control for multiplicity, so findings will be interpreted with caution.

### Conclusion

This study is expected to contribute to new understandings on how communities recognize and perceive skin symptoms, with special reference to early-stage leprosy. It will also offer perspectives on how well DEDIKASI-app is received and how effective it is in enhancing awareness among both the community and health care professionals with regard to identifying leprosy and other common skin problems.

## Abbreviations

CHC: community health center
HCW: health care worker
mHealth: mobile health
NTD: neglected tropical disease
TAM: technology acceptance model
